# Major Histocompatibility Class II Pathway Is Not Required for the Development of Nonalcoholic Fatty Liver Disease in Mice

**DOI:** 10.1155/2013/972962

**Published:** 2013-04-24

**Authors:** Gilles Willemin, Catherine Roger, Armelle Bauduret, Kaori Minehira

**Affiliations:** ^1^Department of Physiology, University of Lausanne, Rue du Bugnon 7, 1005 Lausanne, Switzerland; ^2^Center for Integrative Genomics, University of Lausanne, 1010 Lausanne, Switzerland; ^3^Nestlé Research Center, Vers-chez-les-Blanc, 1000 Lausanne 26, Switzerland

## Abstract

Single-nucleotide polymorphisms within major histocompatibility class II (MHC II) genes have been associated with an increased risk of drug-induced liver injury. However, it has never been addressed whether the MHC II pathway plays an important role in the development of nonalcoholic fatty liver disease, the most common form of liver disease. We used a mouse model that has a complete knockdown of genes in the MHC II pathway (MHCII^Δ/Δ^). Firstly we studied the effect of high-fat diet-induced hepatic inflammation in these mice. Secondly we studied the development of carbon-tetra-chloride- (CCl_4_-) induced hepatic cirrhosis. After the high-fat diet, both groups developed obesity and hepatic steatosis with a similar degree of hepatic inflammation, suggesting no impact of the knockdown of MHC II on high-fat diet-induced inflammation in mice. In the second study, we confirmed that the CCl_4_ injection significantly upregulated the MHC II genes in wild-type mice. The CCl_4_ treatment significantly induced genes related to the fibrosis formation in wild-type mice, whereas this was lower in MHCII^Δ/Δ^ mice. The liver histology, however, showed no detectable difference between groups, suggesting that the MHC II pathway is not required for the development of hepatic fibrosis induced by CCl_4_.

## 1. Introduction

Major histocompatibility class II (MHC II) pathway plays an important role in immune function. The molecules of MHC II are expressed on the surface of antigen-presenting cells such as macrophages, B cells, and dendritic cells [1]. Once the processed antigen loaded onto MHC II molecules is presented on the surface of the cells, it promotes the CD4+ helper T cell recognition. This results in an immune response including the production of inflammatory cytokines [2]. Although the importance of the pathway has been widely studied in the immune processing, its specific roles on hepatic inflammation and fibrosis have not been clearly understood.

A genome-wide association study identified single-nucleotide polymorphisms (SNPs) in the MHC II pathway in lumiracoxib-treated patients that developed a liver injury [3]. The alleles of the genes (HLA-DRB1, 5, -DQB1, and -DQA1) had a strong association with elevated plasma liver enzymes in patients that developed the liver injury after the lumiracoxib treatment. Lumiracoxib, a selective cyclooxygenase-2 (COX-2) inhibitor, has been used for osteoarthritis and acute pain treatment [4, 5]. The use of this COX-2 inhibitor is correlated with an increased risk of cardiovascular events and an acute hepatotoxicity [6, 7]. However whether the MHC II pathway has been involved in the mechanism of these diseases has not been clarified.

A similar observation has been reported with a treatment of amoxicillin-clavulanate, an antimicrobial agent [8, 9]. Again the SNPs in the region around HLA-DRB1 and HLA-DQB1 showed a strong association with the drug-induced liver injury. Interestingly, the alleles in HLA-DQB1 locus were also strongly associated with primary biliary cirrhosis [10], which is the most common autoimmune liver disease. During the development of biliary cirrhosis, T lymphocytes play an important role [11, 12], and a link between the T lymphocytes hyperactivity and drug-induced liver injuries has been recently suggested [13]. In addition, several studies have demonstrated that the genes in the MHC II pathway were significantly upregulated in porcine-serum-induced hepatic fibrosis in rats [14, 15]. These facts imply a strong influence of the MHC II pathway on the susceptibility to develop liver diseases.

Nonalcoholic fatty liver disease (NAFLD) is one of the major liver diseases in industrialized countries. Data are suggesting a strong increase of NAFLD prevalence in the next decades [16, 17]. The disease consists of a diverse spectrum of liver pathologies, starting by hepatic steatosis and then steatohepatitis, a state of hepatic inflammation [18]. Then it progresses toward hepatic fibrosis, cirrhosis, and hepatic carcinoma. Although some molecular pathways that lead to hepatitis in NAFLD can also be activated in the drug-induced liver injuries, the implication of MHC II pathway has not been clearly understood in NAFLD model. Given the repeated reports on the association between alleles on MHC II genes and the susceptibility to a liver inflammation [3, 8–10], this pathway might play an important role in the development of NAFLD.

In the present study, we addressed whether the MHC II pathway might be required for hepatitis development and fibrosis formation. To this end, we chose to use a mouse model lacking all conventional genes in MHC II pathway [19]. The entire MHC II region (80 kb) was deleted, resulting in the removal of the genes encoding the MHC II pathway (H2-A*β*, -A*α*, -E*β*, -E*β*2, and -E*α*). The mouse genes H2-A*β* and -E*β* have the closest homology to the human HLA-DQB1 gene. These mice are viable and fertile without major anatomical or physiological abnormalities [19]. We have studied whether these mice were protected against a high-fat diet-induced hepatitis and a chemical-induced hepatic fibrosis.

## 2. Research Design and Method

### 2.1. Animals, Diet, and Chemicals

B6;129S2-*H*2^*dlAb*1-*Ea*^/J (strain 003584, MHCII^Δ/Δ^) mice in which the 80 kb of MHC II region is deleted were purchased from Jackson Laboratories (Bar Harbor, ME, USA). These mice were bred with C57B6/J mice and the F2 generation was crossbred to obtain MHCII^Δ/Δ^ and wild-type mice. These mice were housed in ventilated cages at an animal facility of the University of Lausanne with 12-hour light and dark cycles and free access to food and water. Five-week-old male mice were subjected to high-fat diet (D12451, Research diets Inc., New Brunswick, NJ, USA) for 4 months. The dietary composition of the high-fat diet was carbohydrates 35%, fat 45%, and protein 20%. Dietary protein was originated from casein, and fat was mainly from lard. All animal procedures used in this study were approved by the Swiss cantonal veterinary service. 

Carbon tetrachloride (CCl_4_), chloroform, methanol, EDTA, sirius red, and aprotinin were purchased from Sigma-Aldrich (Munich, Germany). Kits for measuring plasma alanine transaminase (ALT), aspartate transaminase (AST), and triglycerides (TG) were purchased from Wako Chemicals (Neuss, Germany). Hematoxylin & eosin was purchased from Merck (Geneva, Switzerland). 

### 2.2. High-Fat Diet Experiment

Five-week-old male MHCII^Δ/Δ^ mice and wild-type mice were assigned into the high-fat diet described above. Body weight was measured every month. A glucose tolerance test was performed around the 15th week of the intervention. Mice were fasted for 4 hours and a solution of 1 g of glucose per kg of mouse was injected intraperitoneally. Glycemia was monitored at 0, 15, 30, 60, and 120 min using a glucometer (Bayer, Zurich, Switzerland). 

At the end of the 4-month experiment, mice were fasted for 4 hours and glycemia was measured by a glucometer. Blood was collected by an intracardiac puncture and placed into a tube containing EDTA and aprotinin (2 mM and 0.1-0.2 TIU, respectively) on ice. The plasma was then separated by centrifugation and stored at −80°C until analysis. Liver and epididymal adipose tissues were harvested and weighed. The organs were flash-frozen into liquid nitrogen and stored at −80°C until analysis. 

### 2.3. CCl_**4**_ Treatment

To study the development of hepatic fibrosis, 8-week-old wild-type and MHCII^Δ/Δ^ male mice were treated by 1.25 *μ*L/g body weight CCl_4_ (25% in sunflower oil) twice a week during 4 weeks. Another set of mice was treated by sunflower oil as control vehicle at the same time. Twenty hours following the last treatment, mice were fasted for 4 hours and anesthetized for a cardiac puncture. After sacrifice, blood and liver were kept for further analysis. Fresh liver pieces were put into 4% paraformaldehyde solution during 4 hours. After several washings with PBS, liver samples were dehydrated and parafinated for histological analysis.

### 2.4. Histology

Parafinated liver samples were sliced (4 *μ*m) and stained according to a standard technique with hematoxylin and eosin (H&E) using routine methods. To study the fibrosis formation, 0.1% sirius red was used to stain collagen I, III, and bile pigment using a protocol described elsewhere [20]. For the histological samples, photos were taken with an AXIO Imager M1 with fluo-Axiocam MRm and color Axiocam MRc cameras (Carl Zeiss AG, Oberkochen, Germany). Images were treated by Axiovision release 4.8.2. We then quantified the fibrotic area by assessing the ratio of the red-stained area (fibrosis) to the total area, the vascular luminal area being subtracted if present, using Photoshop (Adobe Systems, Mountain View, USA) in five images per section of the sample (10 x magnification).

Immunohistochemistry by glial fibrillary acidic protein (GFAP) stain was performed by the subsequent incubations of the slices with rabbit anti-GFAP primary antibody (Dako, Glostrup, Denmark) diluted in normal goat serum for overnight at 4°C, then with goat anti-rabbit coupled with Alexa Fluor 568 (Life Technologies, Carlsbad, USA) for 30 minutes in the dark at room temperature. Immunostained sections were then counterstained with 4′,6-diamidino-2-phenylindole (DAPI), embedded with mowiol and analyzed using the Zeiss microscope.

### 2.5. Plasma Parameters and Liver Lipids Analysis

Total plasma ALT and AST were measured by the Roche/Hitachi 912 instrument with the commercial kits mentioned earlier. Insulin was measured by a Mouse Insulin ELISA kit (Mercodia, Uppsala, Sweden). Plasma active Plasminogen activator inhibitor-1 (PAI-1) was also measured by an ELISA kit (Molecular Innovations Inc., Novi).

From the harvested liver samples, total lipids were extracted using a modified Folch method [21, 22]. For the measurement of hepatic TG content, total lipid extract was subjected to SPE columns (Interchim, Montluçon, France) to separate TG [23, 24]. TG were then mixed with a chloroform-triton X (1%) solution and dried under N_2_ gas. TG were thus dissolved into water and the content of TG was measured by the use of the Wako Chemical kit.

### 2.6. Genotyping and Real-Time Polymerase Chain Reaction (PCR)

Ear DNA was extracted by the hotSHOT protocol [25]. Genotyping was performed according to Jackson laboratory's protocol (http://jaxmice.jax.org/strain/003584.html). Briefly, 2 sets of primers were used for the mutated gene (oIMR1020: 5′-Cgg AAg TgC TTg ACA TTg g-3′, oIMR1021:5′-gTA TTg ACC gAT TCC TTg Cg-3′) and wild-type gene (oIMR1273; 5′-AAC CTT CAg gAT CTg TgA TCC-3′, oIMR1274; 5′-gTg gCT gTT gCC TTA AgA CC-3′). After PCR cycles, samples were loaded onto 2% agarose gels and the mutated band (209 bp) as well as the wild-type band (178 bp) was monitored.

Total RNA was extracted from tissues according to the phenol-chloroform extraction protocol in Tri Reagent (Molecular Research Center, Inc., Cincinnati, USA) [26]. cDNA was created by a reverse transcription of 2 *μ*g of total RNA using Superscript II Transcriptase from Invitrogen (Life Technologies, Carlsbad, USA) according to the manufacturer's protocol. Real-time RT-PCR was performed on Applied Biosystems' 7000 Sequence Detection System (Life Technologies, Carlsbad). Twenty-time diluted cDNA samples and 0.3 *μ*M forward and reverse primers (Microsynth AG, Balgach, Switzerland) in a final 10 *μ*L volume were reacted in the following PCR cycle conditions: 10 minutes at 95°C followed by 40 cycles of 15 seconds at 95°C and 1 minute at 60°C. Each sample was analyzed in duplicate using the DeltaDeltaCt method [27]. *β*2 microglobulin (*β*2M) was used as a housekeeping gene to normalize the expression of each gene.

### 2.7. Statistics

All data are shown in mean ± standard error of the mean (SEM). Two groups were compared by Student *t*-test. Four groups were compared by one-way analysis of variance (ANOVA) and once it reached the significance (*F* < 0.05), a post hoc test (Tukey Kramer HSD test) was performed. The statistical analyses were performed by use of the JMP software (SAS Institute Inc., Cary).

## 3. Results

### 3.1. Validation of the Mouse Model

**Figure 1:**
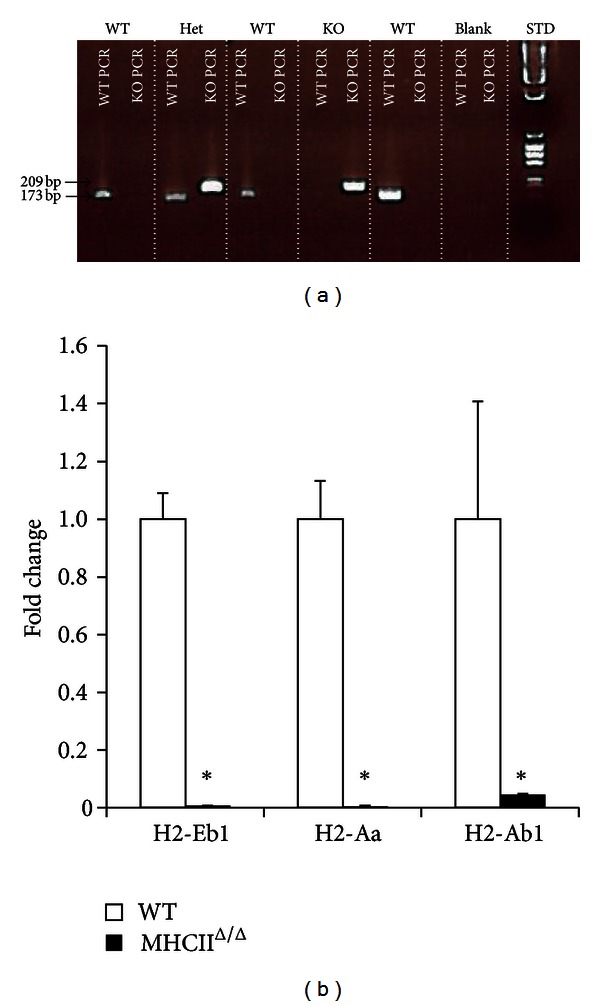
MHCII gene genotyping and hepatic expression in wild-type and MHCII^Δ/Δ^ mice. (a) Two different PCRs were performed for the detection of wild-type (WT PCR) and knockout (KO PCR: MHCII^Δ/Δ^) fragments; 173 bp for a WT band and 209 bp for a KO band. Therefore heterozygous (Het) presents both bands. (b) Gene expression of MHC II genes (H2-Eb1, -Aa, -Ab1) in liver samples by real-time RT-PCR. The expression was normalized by *β*2 microglobulin and compared to the wild-type. Open bars represent wild-type and closed bars MHCII^Δ/Δ^ mice (*n* = 10–14/group). Data are presented as mean ± SEM. *Significantly different from wild-type mice, *P* < 0.05 (Student *t*-test).

The MHCII^Δ/Δ^ mice were originally created by the laboratory of Dr. Christophe Benoist (Institut de Génétique et de Biologie Moléculaire et Cellulaire, Illkirch, France). These mice lack the major genes of MHC II pathway and present very low counts of CD4+ T lymphocytes in the thymus and spleen [19]. The detailed genetic modification and their phenotype have been published [19]. These mice have been backcrossed with C57B6/J strain at Jackson laboratory. After purchasing the mice from Jackson laboratory, we crossed them with C57B6/J mice to obtain control wild-type animals. In this model, the PCR for detecting the knockout allele showed a clear band at 209 bp. The quantitative PCR analysis showed no or very low expression of the genes of the MHC II pathway (Figure 1).

### 3.2. Effect of High-Fat Diet on Inflammatory Status in MHCII^Δ/Δ^ Mice

**Figure 2:**
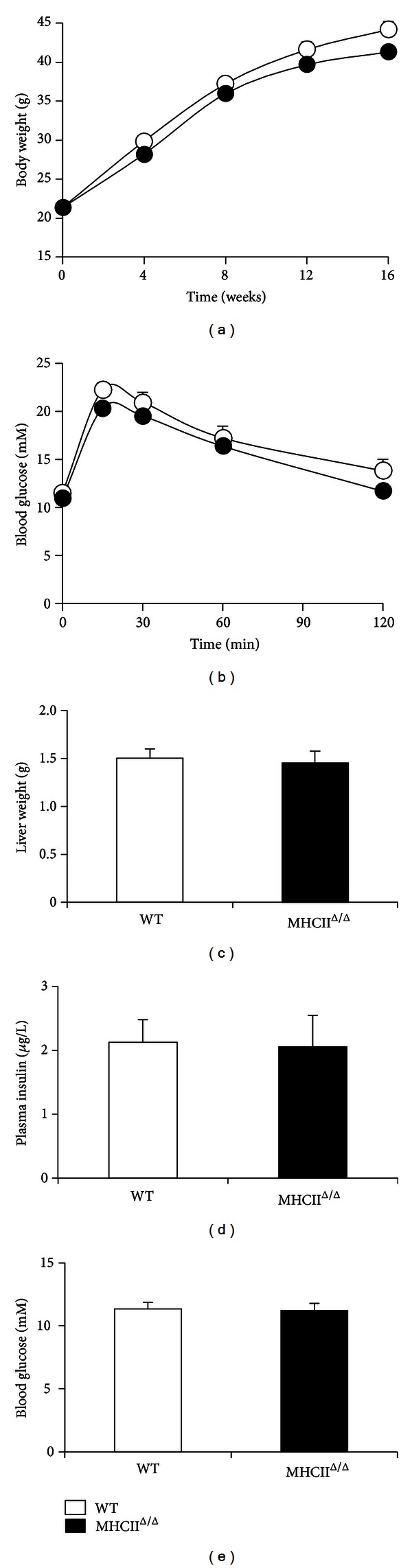
Effect of high-fat diet on metabolic parameters in wild-type and MHCII^Δ/Δ^ mice. Open circle/bars represent wild-type and closed circle/bars MHCII^Δ/Δ^ mice (wild-type: *n* = 25, MHCII^Δ/Δ^: *n* = 20). Data are presented as mean ± SEM. (a) Body weight gain during 4-month diet. (b) Glucose tolerance test was performed around the 15th week of the intervention. (c) Liver weight at 16th week. (d) Fasting plasma insulin concentration at 16th week. (e) Fasting blood glucose concentration at 16th week.

**Figure 3:**
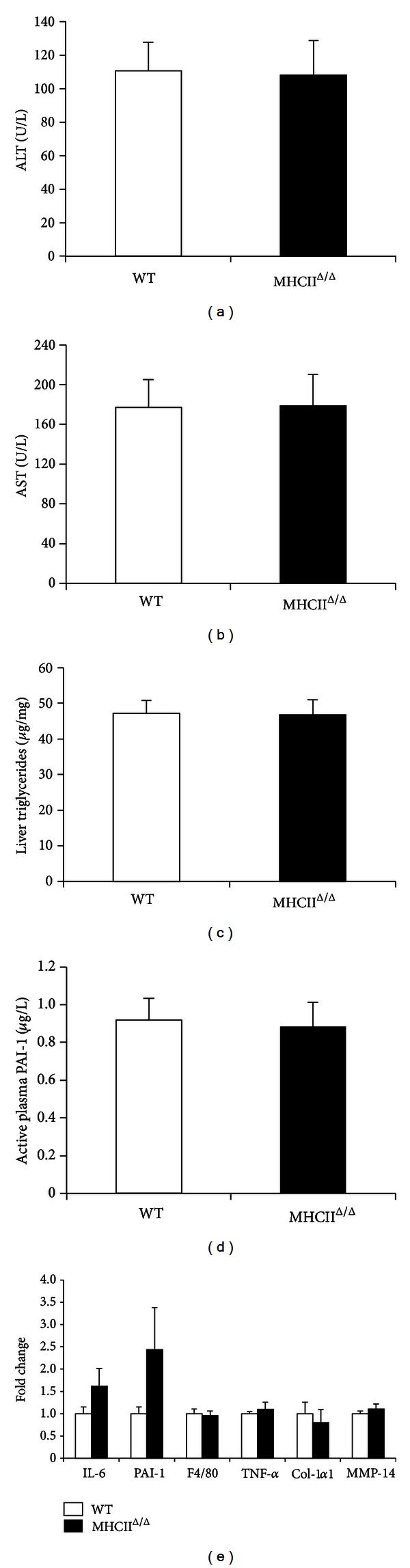
Effect of high-fat diet on liver enzymes, lipids, and inflammatory markers in wild-type and MHCII^Δ/Δ^ mice. Open bars represent wild-type and closed bars MHCII^Δ/Δ^ mice (wild-type: *n* = 25, MHCII^Δ/Δ^: *n* = 20). Data are presented as mean ± SEM. (a) Plasma ALT level at 16th week. (b) Plasma AST level. (c) Liver triglyceride content at 16th week. (d) Fasting active plasma PAI-1 concentration at 16th week. (e) Gene expression related to inflammation and fibrogenesis in the liver at 16th week.

**Figure 4:**
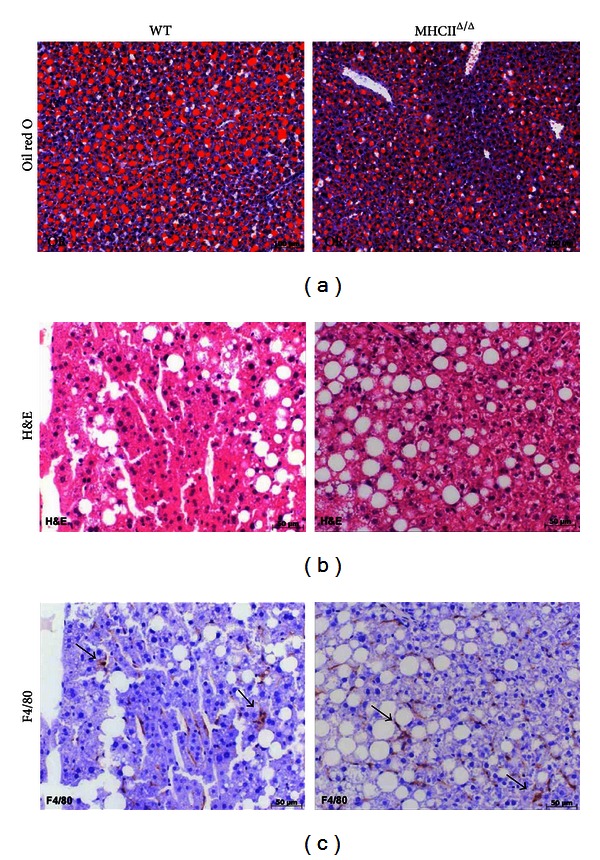
Effect of high-fat diet on liver histology in wild-type and MHCII^Δ/Δ^ mice. (a) Oil red O staining (red color represents the lipid accumulation). (b) Hematoxylin and eosin (H&E) staining. (c) F4/80 immunohistochemistry (macrophages were stained in brown: arrows).

Both wild-type and MHCII^Δ/Δ^ mice similarly gained body weight after 4-month high-fat diet (Figure 2). Liver weight was also similar in both groups. Plasma glucose and insulin concentrations were comparable between groups. As expected, both groups had similar glucose tolerance after the long-term high-fat diet. The liver TG content was also comparable between groups (Figure 3). Oil red O staining confirmed this result (Figure 4). Plasma ALT and AST levels were equally high after the high-fat diet in two groups (Figure 3), suggesting similar liver functions after the high-fat diet.

To study the inflammatory status of the liver after the long-term high-fat diet feeding, we have performed H&E staining and F4/80 for macrophages staining. We observed positive markers of F4/80 and similar lipid droplet morphology in both groups (Figure 4). The expression of genes related to inflammation such as IL-6, TNF-*α*, PAI-1, and F4/80 was not different between groups (Figure 3). The mRNA levels of fibrosis markers such as collagen type 1*α*1 (Col-1*α*1) and matrix metalloproteinase-14 (MMP-14) were also comparable. We also measured active PAI-1 concentration in the plasma (Figure 3). Again no difference was observed between wild-type and MHCII^Δ/Δ^ mice. All together, blocking the MHC II pathway did not affect the hepatic inflammation induced by the high-fat diet in mice.

### 3.3. Effect of CCl_**4**_ Injection on the Formation of Hepatic Fibrosis in MHCII^Δ/Δ^ Mice

**Figure 5:**
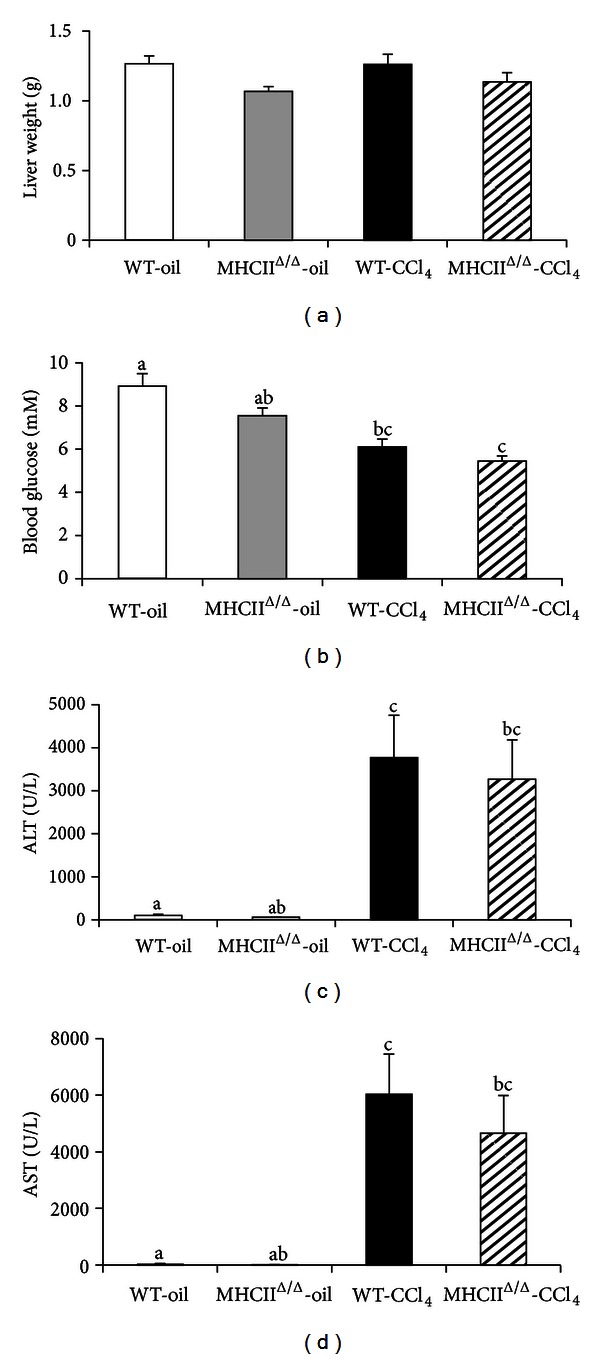
Effect of 4-week CCl_4_ treatment on liver and blood glucose in wild-type and MHCII^Δ/Δ^ mice. Open bars represent oil-treated wild-type (WT-oil), closed bars CCl_4_-treated wild-type (WT-CCl_4_), gray bars oil-treated MHCII^Δ/Δ^ mice, and semiclosed bars CCl_4_-treated MHCII^Δ/Δ^ mice (wild-type oil: *n* = 7, MHCII^Δ/Δ^ oil: *n* = 5, wild-type CCl_4_: *n* = 11, MHCII^Δ/Δ^ CCl_4_: *n* = 9). Data are presented as mean ± SEM. (a) Liver weight. (b) Fasting blood glucose decreased after the CCl_4_ treatment in both groups. (c), (d) Strong increase in fasting plasma ALT and AST was observed after the CCl_4_ treatment. ^a, b, c^ *P* < 0.05, ANOVA and Tukey Kramer HSD test. Different letters indicate the significant difference between groups.

**Figure 6:**
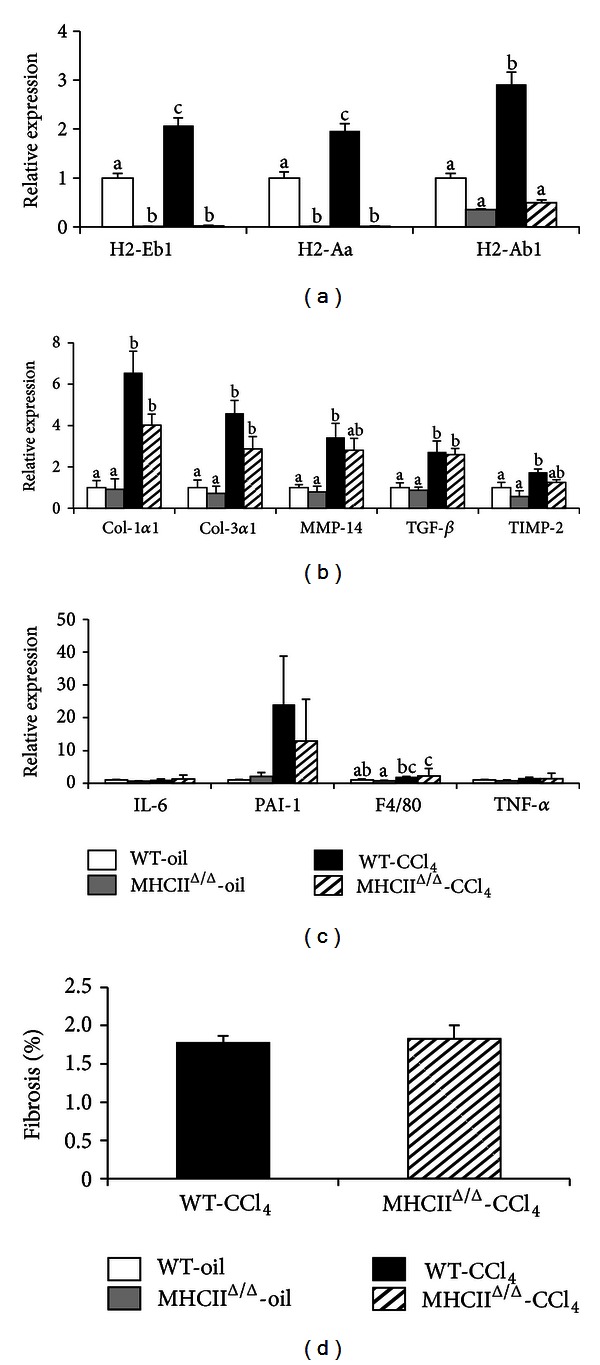
Effect of 4-week CCl_4_ treatment on hepatic gene expression and fibrosis in wild-type and MHCII^Δ/Δ^ mice. Open bars represent oil-treated wild-type (WT-oil, *n* = 7), closed bars CCl_4_-treated wild-type (WT-CCl_4_, *n* = 11), gray bars oil-treated MHCII^Δ/Δ^ mice (MHCII^Δ/Δ^-oil, *n* = 5), semiclosed bars CCl_4_-treated MHCII^Δ/Δ^ mice (MHCII^Δ/Δ^-CCl_4_, *n* = 9). Data are presented as mean ± SEM. (a) MHC II gene expression. (b) Gene expression related to fibrosis formation. (c) Gene expression related to inflammation. (d) No difference in fibrosis (%) between groups treated by CCl_4_. The percentage was determined based on the sirius red staining (5 pictures/mice, WT-CCl_4_: *n* = 5, MHCII^Δ/Δ^-CCl_4_: *n* = 8). ^a, b, c^ *P* < 0.05, ANOVA and Tukey Kramer HSD test. Different letters indicate the significant difference between groups.

We next studied the development of hepatic fibrosis. To this end, we treated wild-type and MHCII^Δ/Δ^ mice with CCl_4_ during 4 weeks. The treatment resulted in a significant decrease of glycemia and a significant increase of ALT and AST in both groups (Figure 5). No difference in liver weight was observed. The CCl_4_ injections also highly induced the expression of MHC II genes in wild-type mice, while MHCII^Δ/Δ^ mice had no induction of these genes, as expected (Figure 6). Genes related to the fibrosis formation such as Col-1*α*1, Col-3*α*1, and transforming growth factor-*β* (TGF-*β*) significantly increased in both groups after the CCl_4_ injections compared to the oil-injected groups. The genes such as MMP-14 and tissue inhibitor of metalloproteinase-2 (TIMP-2) were also highly induced after CCL_4_ treatment in wild-type mice: however, the induction was somewhat blunted in MHCII^Δ/Δ^ mice. 

**Figure 7:**
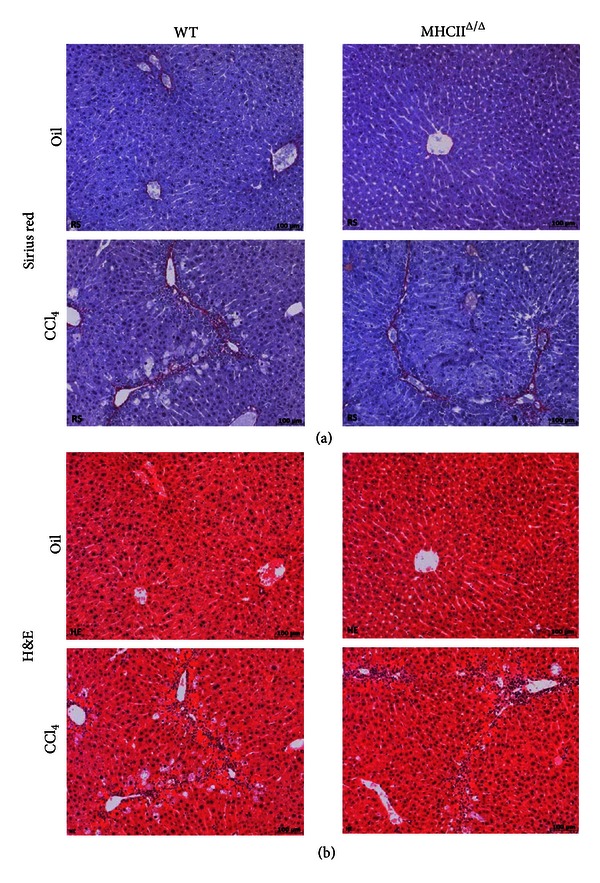
Effect of 4-week CCl_4_ treatment on liver histology in wild-type and MHCII^Δ/Δ^ mice. (a) Sirius red staining for collagen. The red staining represents fibrotic area. (b) Hematoxylin and eosin (H&E) staining.

**Figure 8:**
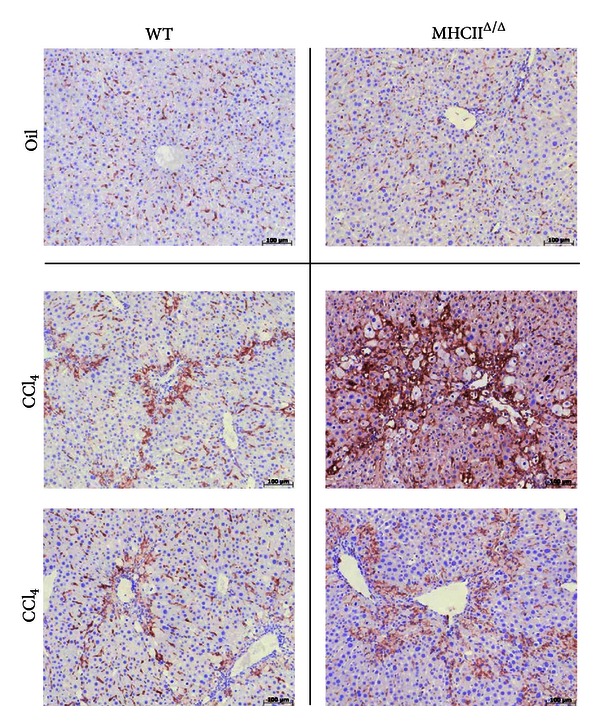
Effect of 4-week CCl_4_ treatment on macrophage staining in wild-type and MHCII^Δ/Δ^ mice. Macrophage staining (F4/80) showed a strong inflammation in both groups treated by CCl_4_.

**Figure 9:**
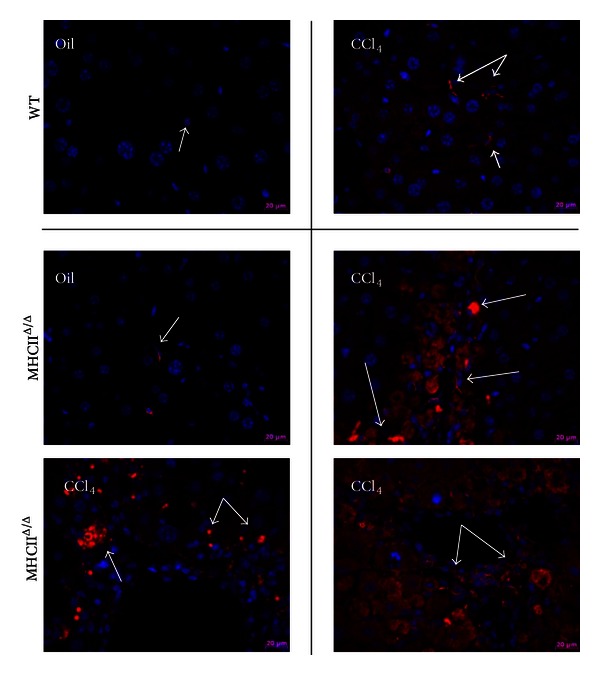
Effect of 4-week CCl_4_ treatment on GFAP staining in wild-type and MHCII^Δ/Δ^ mice. Glial fibrillary acidic protein (GFAP; white arrows) was stained in red. Increased positive staining was observed in CCl_4_-treated MHCII^Δ/Δ^ mice. Blue: DAPI staining.

The liver histology was analyzed using sirius red staining for fibrosis formation. No positive staining was detected in oil-treated groups. The level of positive staining in CCl_4_-treated livers was quantified using Photoshop. We detected a comparable level of fibrosis between groups treated by CCl_4_ (Figures 6 and 7). H&E staining also showed inflammatory signs in the liver treated by CCl_4_ (Figure 7). Again, no remarkable difference was observed between wild-type and MHCII^Δ/Δ^ mice. The staining by F4/80 showed a remarkable infiltration of macrophages in the livers of mice treated by CCl_4_, but, again, no difference was observed between wild-type and MHCII^Δ/Δ^ mice (Figure 8). We further stained stellate cells by GFAP. Interestingly we detected only one positive staining out of 13 in wild-type mice while we found 7 positive stainings out of 9 samples in MHCII^Δ/Δ^ mice (Figure 9).

## 4. Discussion

The present study demonstrated that the MHC II pathway is not implicated in the development of hepatitis when induced by a long-term high-fat diet. We had taken the approach of the high-fat diet to mimic our obesogenic lifestyle that is known to contribute to the nonalcoholic steatohepatitis (NASH). It is also strongly supported that feeding laboratory animals with high-caloric diets such as a high-fat diet can induce NASH [28–30]. As proposed in the pathophysiology of NASH, the “two-hit theory” could also explain the progression of hepatic steatosis to NASH in our experimental model. The first hit is an infiltration of fat into the hepatocytes (hepatic steatosis) and the second hit is characterized by an infiltration of immune cells such as monocytes and lymphocytes because of abnormal oxidative stress [31, 32]. Our mice having undergone a 4-month high-fat diet presented a high amount of fat content in the liver in both groups (first hit). Extensive high-fat diet feeding then resulted in a comparable increase of hepatic inflammation/damage in two groups, judged by macrophage infiltration (second hit). These data suggest that the long-term high-fat diet induced both hepatic steatosis and NASH; however, blocking the MHC II pathway had no influence on the development of hepatic steatosis and NASH in these mice. 

Our long-term high-fat diet also increased the level of ALT and AST in both groups compared to the normal range of ALT and AST generally observed in chow-fed mice (15–80 U/L and 40–120 U/L, respectively, in our laboratory measurements). This result strongly suggests that the modification in MHC II gene expression affected neither ALT nor AST levels, consistent to their comparable liver histology between groups. It has been, however, suggested that some alleles in MHC II pathway were associated with a severity of NAFLD and ALT, but not AST levels, in a Turkish population [33]. In this study, the investigators could not assess a presence of NASH in the population. To our knowledge, this study is the only report suggesting an association between the HLA allele and NAFLD in general population. Many other studies have shown a connection between the HLA allele and plasma ALT or NASH severity in hepatitis C patients or in patients with a drug-induced liver injury [3, 6, 8, 9, 34, 35]. Therefore, MHC II pathway might have a bigger importance to the development of NASH caused by viral infections or drugs than by diet-induced NAFLD.

Secondly, we focused on the chemically induced fibrosis in mice lacking all conventional MHC II genes. As mentioned before, some HLA alleles are associated with an increased susceptibility to develop a drug-induced liver injury. The CCl_4_ treatment strikingly increased the plasma level of ALT, AST, and the degree of hepatic fibrosis in both genotypes. The genes of MHC II pathway were also highly induced by CCl_4_ treatment in wild-type mice, indicating the upregulation of the pathway upon the treatment. Despite the lack of the upregulation of the pathway, the MHCII^Δ/Δ^ mice equally developed a hepatic fibrosis. These data strongly indicate that the MHC II pathway is not required for the development of hepatic fibrosis, at least in the mouse model of CCl_4_-induced hepatic fibrosis. 

Although the major genes implicated in the formation of fibrosis, namely, Col-1*α*1, Col-3*α*1, and TGF-*β* were similarly upregulated in the groups of mice treated by CCl_4_, the induction of some genes such as TIMP-2 and MMP-14 tended to be lower in the MHCII^Δ/Δ^ mice compared to the wild-type mice. TIMP-2 expression has been observed during the early stages of fibrogenesis induced by a porcine serum [36]. We thought that the deletion of MHC II genes might have affected the progression of fibrosis at an earlier time point. In our study, we had tested different periods of injection of CCl_4_ (2-, 4-, 6-, and 8-week treatment) in these mice. In any condition tested, we did not observe a significant difference in the gene expression related to the fibrosis formation (data not shown). We also confirmed these results by liver histology. This again supports the idea that the MHC II pathway is not interfering with the development of hepatic fibrosis induced by CCl_4_. 

Although histological data suggested that there was no difference in the severity of the fibrosis between groups treated by CCl_4_, we found a profound increase in the hepatic stellate cells (HSC) staining only in the group of MHCII^Δ/Δ^ mice treated by CCl_4_. HSC are nonparenchymal cells in the liver and are known to play an important role in fibrosis and tissue repairing [37]. Upon the quiescent HSC activation, they are converted into myofibroblasts, which are responsible for the production of extracellular matrices [38]. The importance of the HSC activation in fibrogenesis was recently reported by Puche et al. [39]. They have elegantly created a transgenic mouse model whose proliferating HSC were selectively killed. By use of the model, Puche et al. demonstrated that these mice had reduced fibrotic area upon CCl_4_ treatment compared to their genetically controlled counterparts. This signifies the important role of HSC in the development of hepatic fibrosis. 

In our study, despite the hyperactivation of HSC in MHCII^Δ/Δ^ mice, the fibrosis formation was strictly comparable between groups. We do not know the exact cause and effect of this HSC induction in MHCII^Δ/Δ^ mice treated by CCl_4_. We have observed that the expression of the genes of the MHC II pathway was very high in HSC fraction when we separated different cellular types in the liver (hepatocytes, HSC, Kupffer cells, and endothelial cells) (unpublished data). This suggests that the HSC could play an important role for the MHC II reaction. We do not know, however, whether the absence of the pathway in MHCII^Δ/Δ^ mice might have affected the HSC proliferation upon CCl_4_ stimulation. On the other hand, the activation of HSC during the development of fibrosis is suggested to be transient [40]. We did not identify when HSC started to be activated in MHCII^Δ/Δ^ mice during the CCl_4_ treatment. Whether this hyperactivation of HSC has compensated the lack of the MHC II pathway and contributed to the fibrosis formation needs to be further addressed. 

The present study clearly demonstrated that the lack of MHC II pathway affected neither NASH induced by high-fat diet nor fibrosis induced by CCl_4_ in mice. Despite a large number of publications implying the association between the alleles in the MHC II pathway and drug-induced liver injury and hepatitis C in humans, this study clearly indicated that the MHC II pathway is not required in the development of NASH and fibrosis at least in mice.
